# Use of Metabolomics as a Complementary Omic Approach to Implement Risk Criteria for First-Degree Relatives of Gastric Cancer Patients

**DOI:** 10.3390/ijms19030750

**Published:** 2018-03-07

**Authors:** Giuseppe Corona, Renato Cannizzaro, Gianmaria Miolo, Laura Caggiari, Mariangela De Zorzi, Ombretta Repetto, Agostino Steffan, Valli De Re

**Affiliations:** 1 Immunopathology and Cancer Biomarkers Unit, IRCCS-National Cancer Institute, 33081 Aviano, Italy; lcaggiari@cro.it (L.C.); mdezorzi@cro.it (M.D.Z.); orepetto@cro.it (O.R.); asteffan@cro.it (A.S.); 2 Oncological Gastroenterology Unit, IRCCS-National Cancer Institute, 33081 Aviano, Italy; rcannizzaro@cro.it; 3 Oncology B Unit, IRCCS-National Cancer Institute, 33081 Aviano, Italy; gmiolo@cro.it

**Keywords:** gastric cancer, metabolomics, first degree relatives, biomarkers, early diagnosis, pepsinogen

## Abstract

A positive family history is a strong and consistently reported risk factor for gastric cancer (GC). So far, it has been demonstrated that serum pepsinogens (PGs), and gastrin 17 (G17) are useful for screening individuals at elevated risk to develop atrophic gastritis but they are suboptimal biomarkers to screen individuals for GC. The main purpose of this study was to investigate serum metabolomic profiles to find additional biomarkers that could be integrated with serum PGs and G17 to improve the diagnosis of GC and the selection of first-degree relatives (FDR) at higher risk of GC development. Serum metabolomic profiles included 188 serum metabolites, covering amino acids, biogenic amines, acylcarnitines, phosphatidylcholines, sphingomyelins and hexoses. Serum metabolomic profiles were performed with tandem mass spectrometry using the Biocrates Absolute*IDQ* p180 kit. The initial cohort (training set) consisted of *n* = 49 GC patients and *n* = 37 FDR. Differential metabolomic signatures among the two groups were investigated by univariate and multivariate partial least square differential analysis. The most significant metabolites were further selected and validated in an independent group of *n* = 22 GC patients and *n* = 17 FDR (validation set). Receiver operating characteristic (ROC) curves were used to evaluate the diagnostic power and the optimal cut-off for each of the discriminant markers. Multivariate analysis was applied to associate the selected serum metabolites, PGs, G17 and risk factors such as age, gender and *Helicobacter pylori* (*H. pylori*) infection with the GC and FDR has been performed and an integrative risk prediction algorithm was developed. In the training set, 40 metabolites mainly belonging to phospholipids and acylcarnitines classes were differentially expressed between GC and FDR. Out of these 40 metabolites, 9 were further confirmed in the validation set. Compared with FDR, GC patients were characterized by lower levels of hydroxylated sphingomyelins (SM(OH)22:1, SM(OH)22:2, SM(OH)24:1) and phosphatidylcholines (PC ae 40:1, PC ae 42:2, PC ae 42:3) and by higher levels of acylcarnitines derivatives (C2, C16, C18:1). The specificity and sensitivity of the integrative risk prediction analysis of metabolites for GC was 73.47% and 83.78% respectively with an area under the curve of the ROC curve of 0.811 that improves to 0.90 when metabolites were integrated with the serum PGs. The predictive risk algorithm composed of the C16, SM(OH)22:1 and PG-II serum levels according to the age of individuals, could be used to stratify FDR at high risk of GC development, and then this can be addressed with diagnostic gastroscopy.

## 1. Introduction

The GC is the fourth most common cancer and the second leading cause of cancer-related death worldwide [1]. Despite its decline in the last century, GC remains a major public health issue, with approximately 950,000 new cases diagnosed every year worldwide, of whom about 723,000 die from the disease [2]. GC is a genetically and phenotypically heterogeneous disease usually detected at an advanced stage with a median survival below one year.

The marked geographic variation, time trends and the migratory effect on GC incidence suggest that both genetic and environmental factors are implicated in the etiology. Besides the immutable inherent risk factors such as age, gender, race, the presence of *Helicobacter pylori* (*H. pylori*) infection, tobacco and diet are considered the major causes of GC [3]. However, other factors may also influence GC susceptibility. Different studies have reported a GC aggregation within FDR with a risk of two to 10 times higher than that of the general population. With the exclusion of the rare (<1% of GC) hereditary diffuse gastric cancer (HDGC) condition harboring a *CDH1* gene mutation [4], the observed familial clustering of GC cannot, to date, be explain only on genetic bases. The widespread use of upper endoscopy, an invasive but sensitive test for GC diagnosis, is limited by cost, risk complication and discomfort to patients and its use is indicated only for very high risk or symptomatic individuals.

The five-year survival rate continues to be poor (about 25% of cases) for GC, but where early diagnosis of cancer was confined to the inner lining of the stomach wall, a five-year survival rate of 95% can be reached. The problem of late diagnostics is due to a substantial proportion of patients with an asymptomatic or unspecified GC disease. Ideally, the GC disease should be diagnosed at an early stage by surveillance and management of individuals at high risk for GC. So far, extensive screening programs for GC have been introduced with success in high-risk countries such as Japan and South Korea. In Japan, eradication of *H. pylori* has been used as a first prevention strategy [5]; a secondary prevention strategy focuses on the diagnosis of GC in an early stage by using endoscopy. In some cases, the combination of serum pepsinogens (PGs) concentration and the presence of *H. pylori* antibody (ABC method) has been recommended based on the knowledge that PG concentration reflects the grade of gastric atrophy, a precursor condition for GC development [6]. The ABC method has been used for GC mass screening since 2011 in Japan (Nishitokyo Medical Association). However, this method is still debated because of the lack of satisfactory evidence in decreasing the mortality rates of GC [7]. Therefore, the finding of an efficacious non-invasive triage of FDR at increased risk for GC, that should undergo endoscopic examination remains a challenge for GC surveillance, particularly in low GC incidence geographic areas.

Metabolomics has emerged as a fast and efficient method to identify novel cancer biomarkers that gradually become a complementary technique to genomics and proteomics [8,9]. Metabolomics specifically addresses the simultaneous monitoring of hundreds to thousands of small molecules (metabolites < 1 kDa) from bio-fluids and tissue samples. The metabolomic profile is retained to give a biochemical snapshot of the physiopathological conditions of the cells/tissues resulting from the complex interplay between host genetic and environmental factors.

In this study, specific deficiency of serum sphingomyelins, phospholipids and an excess of acylcarnitines lipids were detected in GC as potential risk biomarkers. The integration of serum metabolomic biomarkers with other risk factors such as age and serum PG-II levels enhanced the diagnostic power of the pepsinogen test allowing a risk stratification of FDR for endoscopic GC examination. These results underline the role of the use of the individual's metabolomic trait to complement the FDR screening for precancerous conditions.

## 2. Results

### 2.1. Individual Characteristics

Demographic and pathological characteristics of GC patients and FDR in the training and validation sets are reported in Table 1, respectively. The training set comprised 49 GC patients and 37 FDR while the validation set included 22 GC patients and 17 FDR. In both these two sets, age and PG-II level differed significantly (*p* < 0.05) in GC patients and FDR while the other clinical and pathological conditions were superimposable. The odds ratio was 1.12 (95% CI 1.05–1.18) for age, and 1.25 (95% CI 1.10–1.42) for PG-II. The loss of data for histological *H. pylori* infection and GC classification is due to the difficulty of accessing tissue samples collected from the external hospital during routine biopsies for GC diagnosis.

### 2.2. Comparison of Serum Metabolomic Profiles of GC and FDR

**Figure 1:**
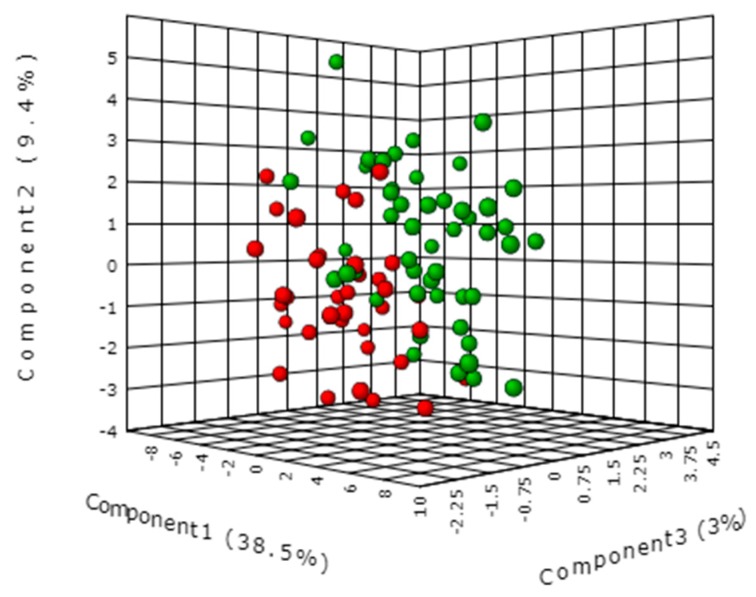
Multivariate partial least squares discrimination analysis (PLS-DA) of serum metabolomic profiles from FDR (red color) and GC patients (green color) in the training set.

The serum targeted metabolomic profiles were investigated by tandem mass spectrometry (MS). The MS-targeted approach for serum metabolomic profile analysis adopted in this investigation has the advantage of being highly robust in term of intra- and inter-day precision and accuracy and overall, its application provides absolute quantification of serum metabolites. All these features contribute to making the targeted approach particularly reliable for clinical metabolomic investigations and guarantees a high standard quality among different clinical laboratories. The list of metabolites analyzed by the targeted metabolomics method used in this study is shown in Supplemental Table S1. Metabolomic profile data were analyzed using supervisor partial least squares discrimination analysis (PLS-DA), which explains maximum separation between GC and FDR samples. The result of this multi-parametric approach was summarized in the PLS-DA graph (Figure 1) where each point corresponds to a metabolomic patient profile. The PLS-DA discriminated GC patients and FDR with a classification accuracy of 72% (*R*^2^ = 0.40, *Q*^2^ = 0.20). Statistical validation of the obtained PLS-DA model was also confirmed with permutation testing (*p* < 0.004). Variable importance in the projection (VIP) of the PLS-DA model indicated that SM(OH)22:1 and SM(OH)22:2 had the higher VIP score (>2.1) (Supplemental Figure S1).

### 2.3. Identification and Selection of the Most Significant Metabolites 

**Figure 2:**
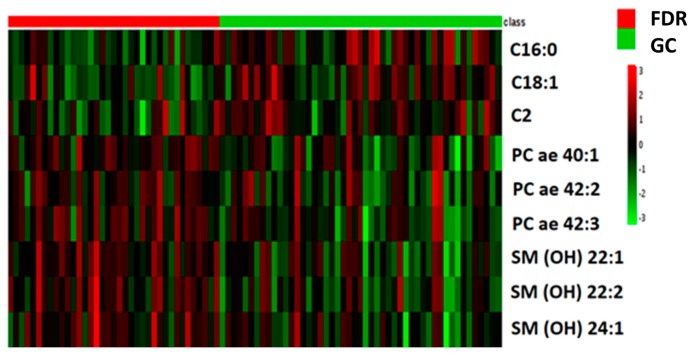
Heat map plot of the differential validated serum metabolites between FDR and GC patients. Data refers to the relative serum concentration level observed in the training set.

**Figure 3:**
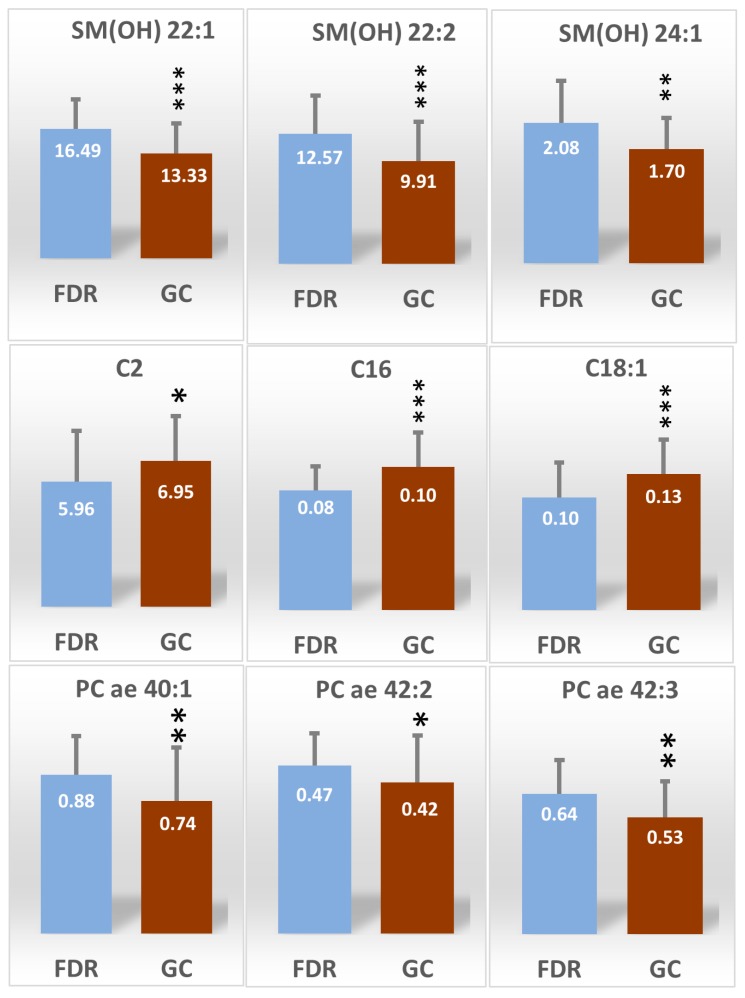
Box plots of the selected and validated differential metabolites (mean ±SD) between FDR and GC patients (training set). For each metabolite the serum concentration mean value is reported inside the box and expressed as μmoles/L. * *p* < 0.05, ** *p* < 0.001, *** *p* < 0.0001.

From the training data set we selected the most significant metabolites that discriminate GC patients and FDR by both univariate (*t*-test, *p* < 0.05) and multivariate analysis (VIP > 1). Forty metabolites met these criteria and are summarized in Table S2. They include carnitine/acylcarnitines (*n* = 7), aminoacid derivatives (*n* = 5), phosphatidylcholines (*n* = 23) and sphingomyelins (*n* = 5) lipids derivatives. Of these 40 metabolites identified in the training set, nine were further confirmed as differentially expressed in the validation set (*p* < 0.05). They were three hydroxylated sphingomyelins: SM(OH)22:1, SM(OH)22:2, SM(OH)24:1, three acylcarnitines: C2, C16 and C18:1 and three phosphatidylcholine lipids PC ae 40:1, PC ae 42:2 and PC ae 42:3. Figure 2 shows the heat map plot of the concentrations of the validated metabolites that show the main significant change between GC patients and FDR in the training set. Hydroxylated sphingomyelins and phosphatidylcholines lipids showed the highest abundance score in FDR, while acylcarnitine’s derivatives presented the lowest abundance score in GC patients. Absolute mean concentration, expressed as serum micromolar concentration, of each validated metabolite in GC patients and FDR are reported in Figure 3. In order to assess the GC’s specificity of these nine validated metabolites, every single level of them was compared with those obtained from patients with non-epithelial cancer (i.e., non-Hodgkin lymphoma (NHL), *n* = 47) and with epithelial cancer (i.e., breast cancer, *n* = 34). When compared with FDR, the levels of the acylcarnitine: C2, C16 and, C18:1 were found to be higher in GC as well as in NHL and breast cancer groups. Conversely, the PC derivatives: PC ae 40:1, PC ae 42:2 and PC ae 42:3 as well as the SM derivatives SM(OH)22:1 and SM(OH)22:2 were found to be lower only in the GC patients (Figure S2, Supplemental Data).

### 2.4. Model Performance for Metabolites

**Figure 4:**
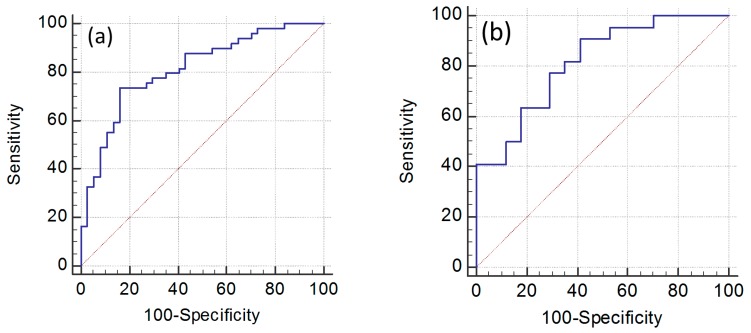
The receiver operating characteristic (ROC) curve plots for the metabolomic model based on C16 and SM(OH)22:1 biomarkers in the training (**a**) and validation set (**b**).

In the multifactor logistic regression model containing the nine established metabolites, high C16 and low SM(OH)22:1 metabolites were found to be independent risk factors for GC patients; in the training set the odds ratio (95% CI) were 2.83 (1.66–4.82) and 1.39 (1.19–1.62) for C16 and SM(OH)22:1, respectively. The equation for logistic regression fit was logit(p) = 0.0778 + 44.76 × C16 − 0.26 × SM(OH)22:1. For the training set, the predictive accuracy of the logistic equation measured by ROC curve analysis gave an area under curve (AUC )of 0.81 (95% CI: 0.75–0.89) with a sensitivity of 73.5% and a specificity of 83.8% (Figure 4a) and an AUC of 0.82 (95% CI: 0.66–0.92) with a sensitivity of 90.9% and a specificity of 59.0% when the same equation was applied to the validation set (Figure 4b). 

### 2.5. Effect of H. pylori Infection on Levels of Sphingomyelins and Acylcarnitines 

**Figure 5:**
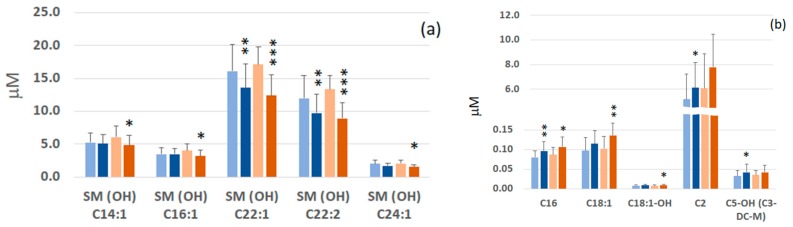
Serum concentrations of significative sphingomyelins phospholipids (**a**) and acylcarnitines derivatives (**b**) in FDR and GC patients according to *H. pylori* infection. Blue and dark-blue columns refer to FDR (*n* = 37) and GC patients (*n* = 44) with negative *H. pylori* infection, respectively. Orange and dark-orange columns refer to FDR (*n* = 14) and GC patients (*n* = 18) with positive *H. pylori* infection, respectively. Statistical comparison performed for FDR vs. GC for both negative and positive *H. pylori* groups by *t*-test: * *p* < 0.05, ** *p* < 0.001, *** *p* < 0.0001.

To investigate the effect of *H. pylori* infection on the observed metabolic differences among GC and FDR, all samples were categorized according to the *H. pylori*-infection status and their sphingomyelins or acylcarnitines serum levels. The mean values of the significative sphingomyelins and acylcarnitines metabolites according to *H. pylori* infection status are shown in Figure 5. The main trend in metabolite profile consists in a decrease of SM(OH)22:1 and SM(OH)22:2 levels and an increase of C16 acylcarnitine in both infected and not infected individuals. In addition, a significant decrease of SM(OH)14:1, SM(OH)16:1 and SM(OH)24:1 and an increase in C18:1 acylcarnitine in *H. pylori*-positive GC was observed, while the C2 and C5 acylcarnitines were increased limitedly in negative *H. pylori*-GC.

### 2.6. Age Effect on the Serum Levels of the SM(OH)22:1 and C16 Metabolites

**Figure 6:**
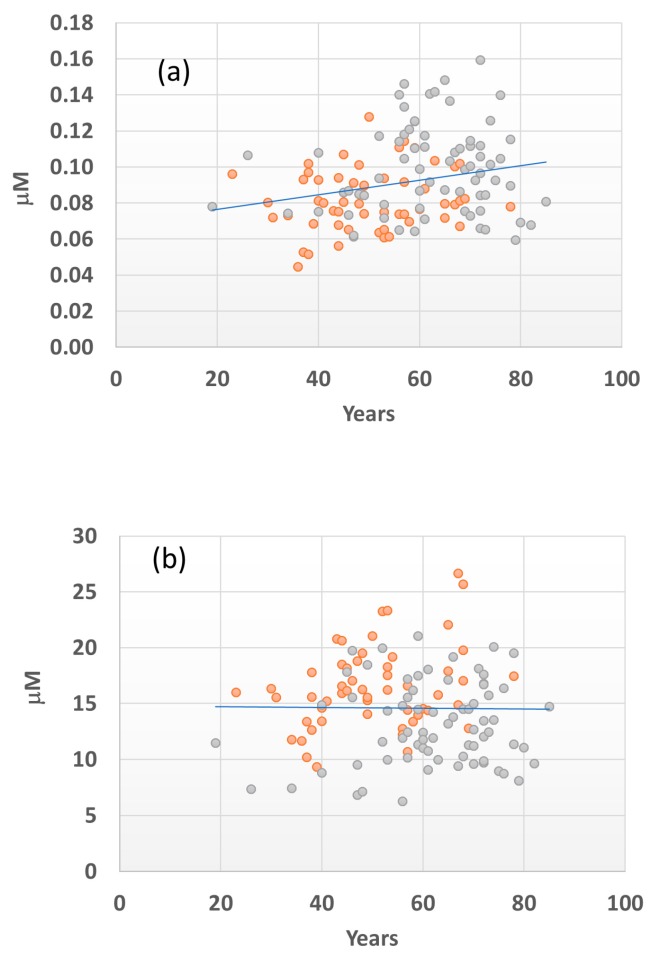
Correlation between C16 (**a**) and SM(OH)22.1 (**b**) serum concentrations and age for FDR (orange) and GC patients (gray). Correlation is performed considering all FDR and GC data. Spearman’s rank test < 0.05 is considered significant.

Since there was a significant difference in the age between FDR and GC patients (median age of 53 vs. 61 for the FDR and GC groups, respectively, *p* < 0.001, Table 1), we further investigated the relationship between the level of metabolites SM(OH)22:1 and C16 and the age of individuals. Considering all the FDR and GC data from both the training and validation sets a significant positive correlation (Spearman’s rank test, *p* = 0.0076) was found between the level of C16 metabolite and age (Figure 6a). Conversely, no significant relationship between the SM(OH)22:1 metabolite and age (Figure 6b) was reported. However, within the single FDR and GC groups, any significant correlation (*p* = 0.4609 and *p* = 0.3081 respectively) was established between the levels of C16 and age.

### 2.7. Integrated Metabolomics Model

Data integration of metabolomics GC signatures with PGs, G17, *H. pylori* infection and individual’s clinical data was our ultimate goal. Association of (a) SM(OH)22:1 and (b) C16 metabolites, and (c) serum PG-II level on GC diagnosis was retained in the multivariate model. Although C16 was found related to age when either FDR and GC group were considered separately, it was found independent by age. Therefore, we included both age and C16 as independent covariates in the logistic classification algorithm. The estimated regression coefficients for markers were as follows: 0.0898 for age, 0.0843 for PG-II, 0.283 for SM(OH)22:1 and 0.604 for C16. Thus, the final equation that stratifies FDR at high risk for GC development is computed as follow:

*Y* = −4.97 + 0.0898 × Age + 0.0843 × PG-II + 0.283 × SM(OH)22:1 + 0.604 × C16

**Figure 7:**
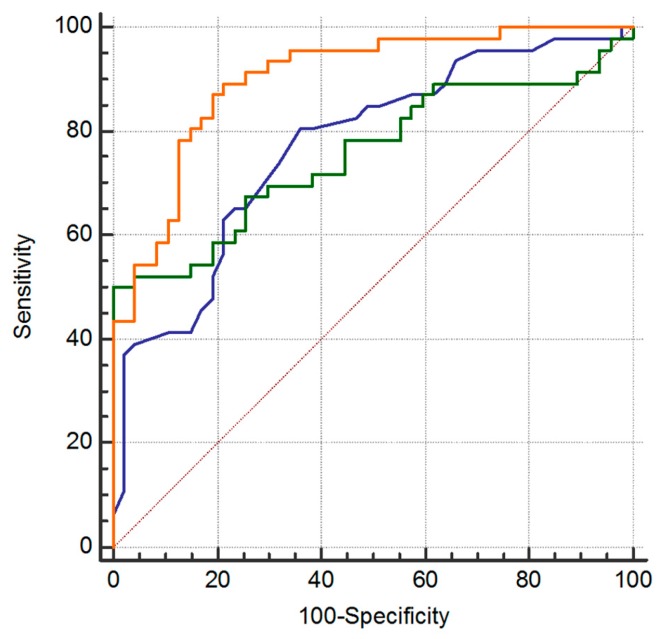
Comparison of ROC curves to test the difference among the areas under 3 dependent ROC curves: patient age (blue), PG-II biomarker (green) and the integrated model including age, PG-II and metabolites (orange).

**Figure 8:**
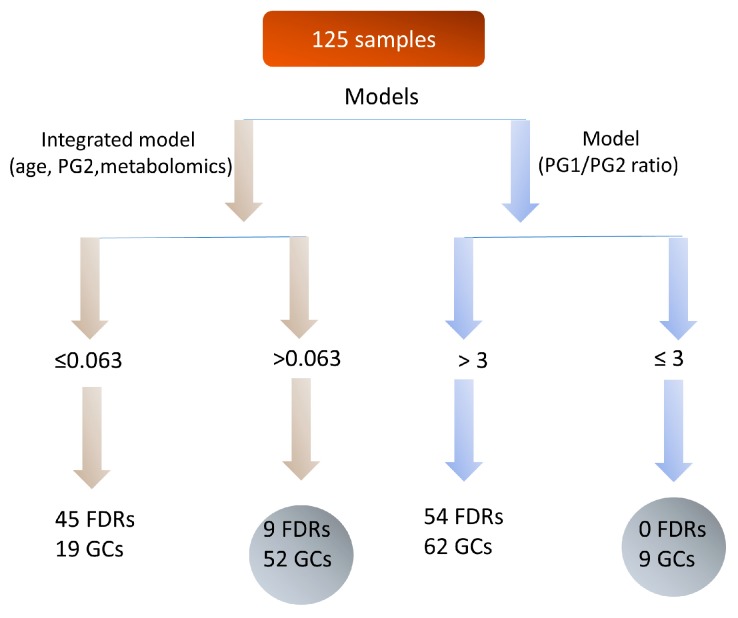
Clinical application of the developed algorithm proposed to identify high risk FDR (*Y*= −4.97 + 0.0898 age + 0.0843 PG-II + 0.283 SM(OH)22:1 + 0.604 C16) compared with the PG-I/PG-II basal model currently in use.

In our series, the equation including the metabolites provided a significantly higher capability of detecting GC than that provided by PG-I/PG-II ratio model. The model achieved good discriminatory power (i.e., AUC = 0.857, 95% CI: 0.78–0.91) (Figure 7). Conversely, the analysis performed with the current screening test for GC based on PG-I/PG-II ratio showed a ROC curve with a lower AUC value of 0.765 (95% CI: 0.67–0.84) (*p* = 0.0278) in our series (Figure 7). The prognostic ability of the model was further evaluated by using the optimal cut-off of *Y* > 0.063, as the score from ROC curve analysis was able to better discriminate FDR from GC patients. By using this cut-off, we correctly identified 52 out of the 71 GC patients (73%). Instead, by using a PG-I/PG-II ratio of ≤3, which is a commonly used cut-off for GC diagnosis, we correctly identified only nine out of the 71 GC patients (12.7%) (Figure 8). The percentage of false-positive cases by using the *Y* > 0.063 cut-off was nine individuals among the 54 FDR with a high-risk profile, while using the *Y* ≤ 3 cut-off for PG-I/PG-II ratio was zero. Individuals identified by the integrated metabolomics/pepsinogen equation have been reported to the gastroenterologist for special attention in the follow-up and, as of now (median follow up of 4 years), they have not developed a GC as confirmed by histological examination of the biopsies. The limited number of FDR (9/54) at higher risk for GC development allows for effective prospective monitoring of these individuals. 

## 3. Discussion

The prognosis of GC remains poor and its early detection is the key factor to improving survival. Screening and prevention programs offer an opportunity to reduce GC mortality, but only a minority of individuals (<1%) shows an identified germline gene defect (i.e., *CDH1* gene mutation), for which intense surveillance or prophylactic gastrectomy are provided. The identification of high risk individuals without *CDH1* mutations for further endoscopic examination to recognize GC at an early stage remains a key point. Only in Japan, where GC incidence is very high, PG-I/PG-II ratio ≤ 3 was used as a screening of GC risk. 

In this study, we explored the potential of a noninvasive screening test for detecting early stage GC in FDR population using a metabolomics tool combined with clinical and pepsinogen tests. Metabolomics is a useful new omic tool to identify specific metabolic dysregulation occurring in GC patients compared with FDR. In our series, the most significant metabolic alterations of GC involve the acylcarnitines, sphingomyelins and phosphatidylcholines pathway. The present study highlighted an integrated model that included sphingomyelin SM(OH)22:1 and acylcarnitine C16 as important risk markers able to identify the large majority of GC (73%). Moreover, among FDR population, a feasible number of high risk individuals (<15%) were identified. These latter FDR have been reported to the gastroenterologist for further endoscopic examination and for special attention follow-up and as of now (median follow-up of 4 years) they have not developed a GC. However, to reach a valid conclusion more time to follow-up is necessary. The integrated model showed a better predictive performance than the PG-I/PG-II ratio test (AUC 0.857 vs. 0.765, respectively). Thus, our data suggested that the herein developed model represents an effective non-invasive test to screen FDR at risk of GC development. The interpretation of findings from this study presents some limitations due to the relatively small sample size and the short time of FDR follow-up as well as potential bias common in retrospective studies. 

Despite these limitations, results provided new molecular insights into the metabolism GC’s hallmarks. The serum metabolites that were found significantly differentiated between GC and FDR appear specific to GC disease. When the FDR levels were compared with those of patients with non-epithelial cancer such non-Hodgkin lymphoma or epithelial breast cancer (Figure S2, Supplementary Data), the acylcarnitines: C2, C16 and C18:1 and were found increased in all patients with cancer, while, the PC ae 40.1, PC ae 42:2 and PC ae 42:3 as well as the SM(OH)22:1 and SM(OH)22.2 decreased in only the GC group, suggesting that these latter phospholipids derivatives are specific to the GC disease.

Acylcarnitines are the obligate cofactors of mitochondrial fatty acid *β*-oxidation. The Acyls-CoA derived from fatty acids are unable to penetrate the mitochondrial outer membrane, but by using carnitine palmitoyltransferase activity, the Acyls-CoA are transformed to acylcarnitines, which are then shuttled into the mitochondrial matrix by carnitine-acylcarnitine translocase. Finally, acylcarnitines are converted back to Acyls-CoA by carnitine palmitoyltransferase 2 localized on the inner mitochondrial membrane. Acyls-CoA then enter into the cycle of citric acid to generate NADH and FADH_2_ to produce ATP along the electron transport chain [10]. An imbalance between the fatty acid uptake and the oxidation due to defects or alterations in mitochondrial respiratory complex activities arises in intracellular concentration of acylcarnitines that may be reflected at the serum level [11]. In our series, C18:1, C18:1(OH) and C16 acylcarnitine levels increased in GC patients according to *H. pylori* infection (Figure 6a), suggesting a positive correlation between these acylcarnitines and the bacterium presence. The oxidative stress is one of the major factors in the development of gastric diseases, while the inflammatory state associated with chronic *H. pylori* infection may increase the risk of GC development due to the continuous exposure to oxidative species. Thus, *H. pylori* infection may partially explain the higher serum level of specific acylcarnitine metabolites shown in our patients. On the other hand, increased acylcarnitines in GC patients may be the consequence of the oxidative stress associated to GC itself or to the higher age of patients since oxidative stress has been reported to increase with aging [12]. Interestingly, we found a positive correlation between C16 acylcarnitine and patient’s age (Figure 6). Thus, it is possible that the higher level of C16 carnitine observed in GC patients may be related to both *H. pylori* infection and the age of the patient.

Conversely to acylcarnitines, some phosphatidylcholine derivatives such as PC ae 40.1, PC ae 42:2 and PC ae 42:3 were significantly lower in the serum of GC patients. The lower level of specific serum phospholipids in GC serum could reflect alterations at tumor tissue. A previous metabolomics investigation, performed by a mass spectrometry imaging technique, revealed that GC tissue as compared with normal gastic mucosa may present specific shortages of phosphatidylcholine lipids derivatives i.e., PC 36:4 and PC 34:2 [13,14]. Interestingly, the authors of this study were able to demonstrate that the supplementation of such phosphatidylcholines in the culture medium suppressed the NIH-3T3 transformation by K-Ras as well as the in vitro growth of 4 out of 8 GC cell lines. Moreover, their oral administration was found to also reduce the in vivo growth of GC cells in nude mice without any side effects [13,14]. Overall, these preliminary results underline the importance of the specific serum phospholipids shortage with the GC growth. The lower serum concentrations of other specific phospholipids belonging to the sphingomyelins class observed in this study further reinforce such suggestions.

Sphingomyelins are structural constituents of all cell membranes particularly abundant in the myelin membrane sheaths surrounding axons. Sphingomyelins interact with cholesterol and glycerophospholipids participating in the formation and maintenance of lipid microdomains in the plasma membrane known as lipid rafts. Sphingolipids in lipid rafts modulate many cell processes, such as membrane sorting and trafficking, cell polarization and signal transduction [15]. Through the action of the sphingomyelinase (SMse), the sphingomyelins play a relevant role also in determining the cell fate by hydrolyzing back to ceramide which is an important metabolic intermediate able to induce cellular apoptosis [16]. Thus, sphingolipids have emerged as key effectors in different tumors such as colon cancer, breast cancer, leukemia, esophagus cancer, and brain cancer [17], by controlling various aspects of tumor cell growth and proliferation through ceramide molecules [15]. The specific metabolic signature observed in GC involved a lower serum level of several 2-hydroxylated sphingomyelins (SM(OH)s): SM(OH)22:1, SM(OH)22:2 and SM(OH)24:1 (Figure 3). Collectively, these specific hydroxylated sphingolipids require the action of the cellular fatty acid hydroxylase (FA2H) for their synthesis [18] and like the other sphingomyelins, they can be hydrolyzed to generate 2 hydroxy-ceramide derivatives, which analogously to ceramides have a pro-apoptotic activity [19]. Thus, a shortage of SM(OH)s sphingolipids may contribute to a reduction in cellular ceramide load promoting cell proliferation and tumor survival. Of interest, many cancers, including stomach, pancreas, and colon, show increased nerve density in relation to tumor growth [20]. Nerves infiltrating the GC microenvironment were found to release neurotransmitters to promote tumor growth and reciprocally, tumors secrete neurotrophic factors, that stimulate both nerve outgrowth and cancer cell growth [21]. The lower levels of circulatory sphingomyelins may reflect the increase tumor nerves growth observed in GC. Taken together, these findings suggest further investigations on whether nerve–cancer cell cross-talk involves sphingolipids in GC.

## 4. Experimental Section

### 4.1. Participants

Participants were excluded from clinically significant medications, surgery, radiotherapy or chemotherapy for metabolic, liver, kidney diseases or any other cancers. From January 2009 to March 2014, 71 GC patients and 54 FDR were consecutively enrolled at the Oncological Gastroenterology, Centro di Riferimento Oncologico, IRCCS-National Cancer Institute, Aviano, Italy to characterize their serum metabolomic profiles. For all the GC patients diagnosis was confirmed histologically based on tissue specimens. For all FDR individuals, the GC lesion was excluded after gastroscopy and histological examination of the biopsies. Two additional cohorts of patients unrelated to GC patients with a representative non-epithelial (i.e., non-Hodgkin lymphoma; *n* = 47) and epithelial cancer (breast cancer; *n* = 34) were included in the metabolomics investigation as unrelated independent cancer groups. None of GC patients and FDR were treated with proton pump inhibitors. *H. pylori* infection was detected in tissue sections using hematoxylin and eosin and Giemsa stains as previously reported [22]. Serum PG-I, PG-II and G-17 levels were measured as previously reported [22]. Clinical data from GC patients and FDR were collected in a dedicated database in the oncological gastroenterology center. Before enrolling each participant gave informed written consent. The study was approved on December 2008 by the Institutional Review Board (ref no. IRB2008-14). 

### 4.2. Sample Collection

All the study participants were in an overnight fasting state and 5 mL of peripheral venous blood was taken in the morning. The blood was then allowed to clot for 30 min at 37 °C water bath and followed by centrifugation at 3000 rpm for 15 min. Then, the serum supernatant was taken and transferred to a clean tube and stored at −80 °C until further analysis.

### 4.3. Design of the Study

Based on the diagnosis, participants were randomly divided into 2 sets; the training set included 49 GC patients and 37 FDR and a validation set of 22 GC and 17 FDR. Detailed characteristics of patients are listed in Table 1.

The main steps of the study were: (1) characterization of metabolomic profiles associated with FDR and GC patients, (2) identification and validation of the most important metabolomics GC signatures by using the independent validation sample set, (3) apply multivariate statistical analysis of selected serum metabolites, clinical data and pepsinogen biomarkers to develop an integrated risk model for early stage GC detection, and (4) performance comparison between the model including the metabolites and the model based on PG-I/PG-II risk score. 

### 4.4. Metabolomics Investigation

A high-throughput liquid chromatography-tandem mass spectrometry (LC-MS/MS) platform has been applied to evaluate serum metabolomics profiles. We used the commercial Absolute*IDQ* p180 Kit (Biocrates Life Sciences, Innsbruck, Austria) according to the manufacturer’s instructions for the quantification of 188 targeted metabolites covering the following compound classes: amino acids, biogenic amines and polyamines (*n* = 40), acylcarnitines (*n* = 40), di-acyl-phosphatidyl lipids (*n* = 92), sphingolipids (*n* = 15) and hexose (*n* = 1). The complete list of all metabolites investigated is reported in supplemental Table S1. The analytical system consisted of a liquid chromatography Agilent (Agilent, Santa Clara, CA, USA) coupled with an ABI4000 triple quadrupole mass spectrometer (ABsciex, Framingham, MA, USA). 

Briefly, 10 μL of serum was loaded onto an inserted filter in a 96-well sandwich plate, which already contained appropriate internal standards, structurally identical but labeled with stable isotopes such as deuterium, ^13^C, or ^15^N. The filters were dried under a nitrogen stream, derivatization of amino acids was performed with 5% phenylisothiocyanate (PTC), and filters were dried again. After extraction of metabolites with 500 μL of 5 mM ammonium acetate in methanol, the solution was passed through a filter membrane and diluted with MS running solvent. Final extracts were then analyzed by LC-MS/MS using amino acids and bioactive amines PTC-derivatives the Zorbax SB 100 × 2.1 mm column (Agilent, Santa Clara, CA, USA), and a direct flow injection analysis (FIA-MS/MS) for the analysis of acylcarnitines and phospholipids. Quantification of metabolites was achieved by multiple reaction monitoring, neutral loss and precursor ion scan in positive and negative ion mode. The MS/MS signals were integrated, by using Analyst 1.6.1 (ABsciex, Framingham, MA, USA) and quantified using a calibration curve according to the AS-180 to the manufacturer’s instructions. Concentration and validation data were then further processed using the MetIQ software by comparing the results of triplicate analysis of low, medium and high-quality serum controls as an integral part of the analytical. 

### 4.5. Data Processing and Statistical Analysis

Prior to statistical analysis, the serum concentration values of metabolites investigated were set to a log scale and auto-scaled (mean-centered and divided by the standard deviation of each variable). A supervisor multivariate partial least squares discrimination analysis (PLS-DA) was then applied to identify the relevant metabolites that contributed the most significance in differentiating between the GC and FDR groups in the training set. The PLS-DA model was further cross-validated by comparison of the resulting goodness of fit (*R*^2^), predictive ability (*Q*^2^) values, and by internal validation using 1000 permutation tests. A variable importance in projection (VIP) score was applied to rank the patients’ metabolites that best distinguished between the GC vs. FDR groups. The relevant metabolites that distinguished the two groups in the training set were selected on the basis of VIP > 1 and by *p* < 0.05 as resulted from the application of univariate *t*-test analysis. The more significant metabolites differentially expressed were further validated by the confirmation of significant variable (*p* < 0.05) in the validation set. The ROC curves were constructed to test the diagnostic performance of the more significant metabolomics biomarkers. In a ROC curve, the true positive rate (sensitivity) was plotted against the false positive rate (1-specificity) for different cut-off points of a given parameter. The ROC curve was validated by internal cross-validation and permutation testing. The optimal cut-off was assessed by jointly maximizing sensitivity and specificity. Sensitivity and specificity, computed at the optimal cut-off, were then used for further investigation. All above data processing and the statistical analysis that included ROC analysis were performed using the Metabolanalyst web portals [23]. Correlation between metabolite biomarkers and clinical features was analyzed by the Spearman’s rank-order correlation test. Multivariate analysis was used to determine the coefficient value for each of the independent variables and to make the integrated model equation including metabolites, patient age and PG-II biomarker.

## 5. Conclusions

This exploratory study describes for the first-time serum metabolomic profiles that discriminate GC patients from FDR sharing the same environment and a similar genetic background. As compared with FDR, the GC patients showed specific serum metabolomic signatures characterized by an increase in specific acylcarnitines and a decrease in a distinctive subclass of sphingolipids. The inclusion of such serum metabolomic signatures with patient age and pepsinogen PG-II demonstrated they are a key factor for the development of a model to distinguish FDR from GC patients. Compared with the current PG-I/PG-II screening approach used in Japan, the model proposed showed an improved discrimination between GC patients and the FDR. The current results demonstrate the potential usefulness of the serum metabolomics as a noninvasive tool for the triage of individuals at higher risk of GC development for further endoscopic examination. The feasibility of this approach, as well as the biochemical mechanisms implicated in GC development, remain to be validated and warrant further investigation.

## Figures and Tables

**Table 1:** GC patients and FDR characteristics in the training set (**a**) and in the validation set (**b**).

**(a)**			
	**GC**	**FDR**	***p ^a^***
*N*	49	37	NS
M/F ^b^	27/22	10/27	NS
Age ^c^	61 (19–85)	53 (30–69)	0.00009
*H. pylori* (−) ^d,#^	32	25	NS
*H. pylori* (+) ^e,#^	14	10	NS
PG-I (ng/mL) ^f^	118.2 (2.7–706.4)	97.2 (3.1–658.4)	NS
PG-II (ng/mL) ^g^	17.2 (1.1–104.0)	9.8 (0.2–35.5)	0.0075
G17 (pmol/L) ^h^	15.7 (0.9–983.0)	3.7 (0.4–109.8)	NS
Histological GC Type ^#^			
Intestinal	17		
Diffuse	11		
Mixed	5		
**(b)**			
	**GC**	**FDR**	***p ^a^***
*N*	22	17	
M/F ^b^	12/10	9/8	NS
Age ^c^	67 (34–79)	45 (23–78)	0.001
*H. pylori* (−) ^d,#^	12	12	NS
*H. pylori* (+) ^e,#^	4	4	NS
PG-I (ng/mL) ^f^	107.5 (3.9–341.2)	87.9 (59.3–112.0)	NS
PG-II (ng/mL) ^g^	12.6 (2.8–45.9)	9.0 (4.5–13.8)	0.033
G17 (pmol/L) ^h^	3.8 (1.5–500.0)	4.0 (0.5–14.6)	NS
Histological GC Type ^#^			
Intestinal	8		
Diffuse	2		
Mixed	1		

^a^ Statistical significance of the differences between GC (gastric cancer) and FDR (First-Degree Relatives) evaluated by *t*-test, ^b^ M: male, F: female, ^c^ age expressed as median and (range), ^d^ non infected *H. pylori* patients, ^e^ infected *H. pylori* patients, ^f,g^ serum pepsinogen I and II concentration, ^h^ gastrin 17 serum concentration. ^#^ data loss is due to no access to tissue samples from the external hospital. NS: not significant.

## Supplementary Materials

The following are available online at http://www.mdpi.com/1422-0067/19/3/750/s1.
