# Supplementary material for: Use of Metabolomics as a Complementary Omic Approach to Implement Risk Criteria for First-Degree Relatives of Gastric Cancer Patients
Source: Int J Mol Sci. 2018 Mar 7;19(3):750. doi: 10.3390/ijms19030750 (PMC5877611; doi:10.3390/ijms19030750)
Supplement: Supplementary file 1 [file ijms-19-00750-s001.pdf]

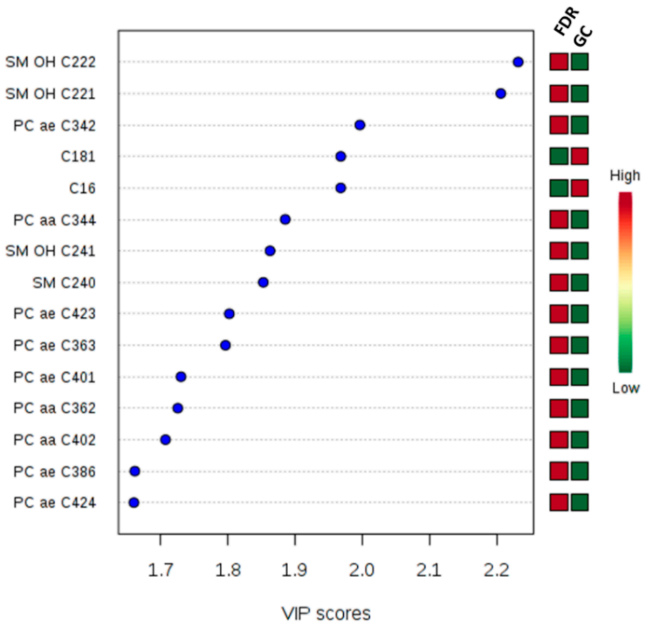

Figure S1. Variable importance in projection (VIP) score plot for the top 15 most important metabolite features identified by PLS-DA. The box indicate the relative concentration from GC and FDR groups.

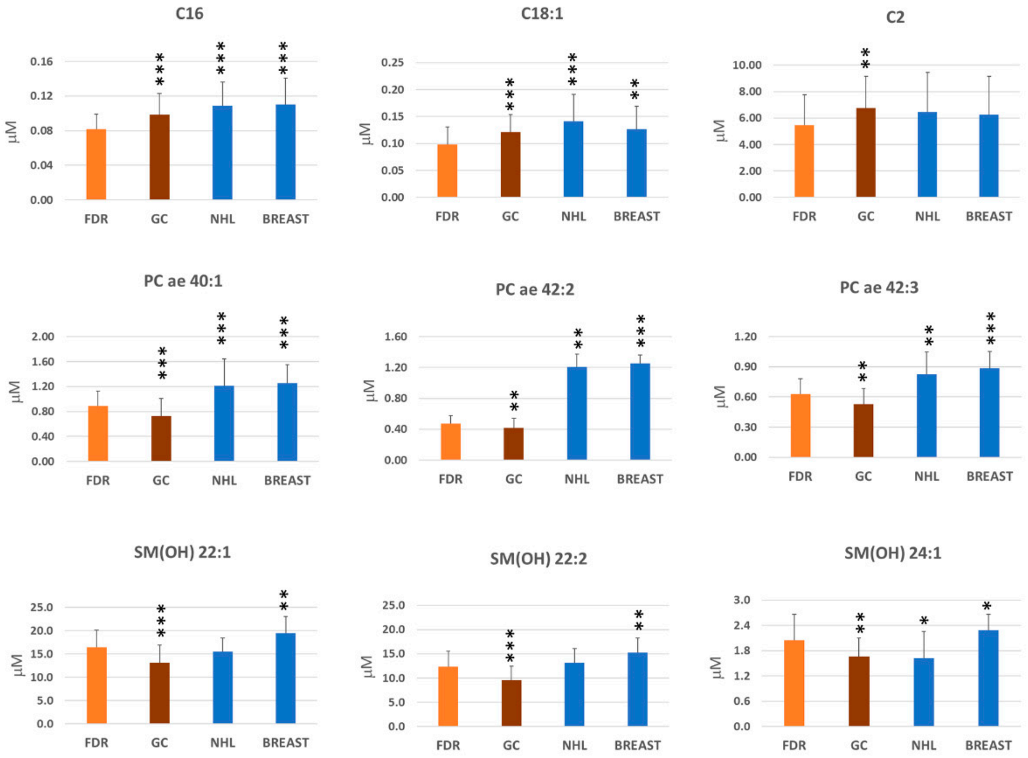

Figure S2. Comparison of metabolites serum concentrations between GC (n = 71), NHL (n = 41) and breast cancer (n = 34) patients as compared with those observed in GC's FDR group (n = 54). Value are expressed as mean ±SD, Statistical significance: * < 0.05, ** < 0.01 and *** < 0.0001 by *t*-text.

Table S1. List of metabolite determined using LC and FIA MS/MS platform

Metabolite	N		
Amino acids	*21*	Ala,Arg,Asn,Asp,Cit,Gln,Glu,Gly,His,Ile,Leu, Lys,Met,Orn,Phe,Pro,Ser,Thr,Trp,Tyr,Val.	*Amino acid metabolism, urea-cycle, activity of gluconeogenesis and glycolysis, insulin sensitivity, neurotransmitter metabolism, oxidative stress*
Biogenicamine	*19*	ADMA, AcOrn, carnosine, creatinine, histamine, kynurenine, Met-SO ,Nitro-Tyr, OH-Pro,PEA,Putrescine,SDMA,sarcosine,serotonin,spermidine,spermine,taurine,alpha-AAA,totalDMA.	*Neurological disorders, cell proliferation, cell cycle progression, DNA stability, oxidative stress*
Carnitine	*1*	*C0*	*Energy metabolism, fatty acid transport and mitochondrial fatty acid oxidation, ketosis, oxidative stress, mitochondrial membrane damage*
Acylcarnitines	*26*	C2, C3, C3:1, C4, C4:1, C5, C5:1, C6 (or C4:1-DC), C6:1, C8, C8:1, C9, C10, C10:1, C10:2, C12, C12:1,C14, C14:1, C14:2, C16, C16:1, C16:2, C18, C18:1,C18:2.	*“*
Hydroxy- and dicarboxyacylcarnitines	*13*	C3-OH, C4-OH (or C3-DC), C5-DC (or C6-OH), C5-OH (or C3-DC-M), C5:1-DC, C5-M-DC, C7-DC, C12-DC, C14:1-OH, C14:2-OH, C16:1-OH, C16:2-OH, C16-OH, C18:1-OH.	*“*
Sum of hexoses	*1*	*H1*	*Carbohydrate metabolism*
Sphingomyelins	*10*	SM 16:0, SM 16:1, SM 18:0, SM 18:1, SM 20:2, SM 22:3, SM 24:0, SM 24:1, SM 26:0, SM 26:1.	*Signalling cascades, membrane damage (eg,neurodegeneration)*
Hydroxysphingomyelins	*5*	SM (OH) 14:1, SM (OH) 16:1, SM (OH) 22:1, SM(OH) 22:2, SM (OH) 24:1.	*“*
Diacyl-phosphatidylcholines	*38*	*PC aa* 24:0/26:0/28:1/30:0/30:2/32:0/32:1/32:2/32:3/34:1/34:2/34:3/34:4/36:0/36:1/36:2/36:3/36:4/C36:5/36:6/38:0/38:1/38:3/38:4/38:5/38:6/40:1/40:2/40:3/40:4/40:5/40:6/42:0/42:1/42:2/42:4/42:5/42:6.	*Degradation of phospholipids, membrane damage, signalling cascades, fatty acid profile*
Acyl-alkyl-phosphatidylcholines	*39*	*PC ae* 30:0/30:1/30:2/32:1/32:2/34:0/34:1/34:2/34:3/C36:0/C36:1/36:2/36:3/36:4/36:5/38:0/38:1/38:2/38:3/38:4/38:5/38:6/40:0/40:1/40:2/40:3/40:4/40:5/40:6/42:0/42:1/42:2/42:3/42:4/42:5/44:3/44:4/44:5/44:6.	*“*
Lyso-phosphatidylcholines	*15*	*lysoPC aa* 6:0/14:0/16:0/16:1/17:0/18:0/18:1/18:2/20:3/20:4/24:0/26:0/26:1/28:0/28:1.	*Degradation of phospholipids (phospholipase activity), membrane damage, signalling cascades, fatty acid profiles*

aa, acyl-acyl; ae, acyl-alkyl;; x:y, where x is the number of carbons in the fatty acid side chain; y is the number of double bonds in the fatty acid side chain; DC, decarboxyl; M,methyl; OH, hydroxyl; PC, phophatidylcholine; SM, sphingomyelin.

Table S2. List of metabolites differential expressed between FDR and GC patients in training set

N	*Metabolite**	*Variation ***	*t.stat*	*p.value*	*log.p*	*q value-FDR*
1	C0	↑	-2.5076	0.014082	1.8513	0.09
2	C14	↑	-2.499	0.0144	1.8416	0.09
**3**	**C16**	↑	**-3.4861**	**7.82E-04**	**3.1068**	**0.023458**
**4**	**C18:1**	↑	**-3.4862**	**7.82E-04**	**3.107**	**0.023458**
**5**	**C2**	↑	**-2.3713**	**0.020016**	**1.6986**	**0.10723**
6	C3	↑	-2.3331	0.022033	1.6569	0.1135
7	C5-OH C3-DC-M	↑	-2.674	0.009005	2.0455	0.070612
8	Cit	↑	-2.3107	0.023303	1.6326	0.1135
9	Creatinine	↑	-2.2745	0.025488	1.5937	0.1135
10	Ile	↑	-2.0629	0.042212	1.3746	0.17113
11	total DMA	↑	-1.9913	0.049703	1.3036	0.18639
12	Thr	↓	2.4174	0.017797	1.7496	0.10291
13	PC aa 32:2	↓	2.667	0.009179	2.0372	0.070612
14	PC aa 34:3	↓	2.3745	0.01985	1.7022	0.10723
15	PC aa 34:4	↓	3.3202	0.001332	2.8754	0.030619
16	PC aa 36:2	↓	3.0067	0.003483	2.4581	0.043537
17	PC aa 36:6	↓	2.4165	0.017838	1.7487	0.10291
18	PC aa 40:2	↓	2.9715	0.003865	2.4129	0.044591
19	PC aa 40:3	↓	1.9976	0.048996	1.3098	0.18639
20	PC aa 42:0	↓	2.0485	0.04363	1.3602	0.17222
21	PC aa 42:2	↓	2.6227	0.010356	1.9848	0.070612
22	PC aa 42:6	↓	2.7928	0.006469	2.1892	0.05708
23	PC ae 32:2	↓	2.2707	0.025726	1.5896	0.1135
24	PC ae 34:2	↓	3.5441	6.46E-04	3.1895	0.023458
25	PC ae 34:3	↓	2.6496	0.009627	2.0165	0.070612
26	PC ae 36:2	↓	2.1312	0.035995	1.4438	0.14998
27	PC ae 36:3	↓	3.1446	0.002299	2.6384	0.034492
28	PC ae 36:4	↓	2.8437	0.005598	2.252	0.052481
29	PC ae 38:0	↓	2.3005	0.023901	1.6216	0.1135
30	PC ae 38:6	↓	2.8841	0.004985	2.3023	0.050255
31	***PC ae 40:1***	↓	***3.0159***	***0.003389***	***2.4699***	***0.043537***
32	***PC ae 42:2***	↓	***2.2926***	***0.024371***	***1.6131***	***0.1135***
33	***PC ae 42:3***	↓	***3.1558***	***0.002222***	***2.6532***	***0.034492***
34	PC ae 42:4	↓	2.8813	0.005026	2.2988	0.050255
35	PC ae 44:6	↓	2.1898	0.031311	1.5043	0.13419
36	SM 16:1	↓	2.6356	0.010001	2	0.070612
37	*SM 24:0*	↓	*3.2555*	*0.001633*	*2.787*	*0.030619*
38	***SM (OH) 22:1***	↓	***3.9828***	***1.44E-04***	***3.8407***	***0.010822***
39	***SM (OH) 22:2***	↓	***4.0385***	***1.18E-04***	***3.9266***	***0.010822***
40	***SM (OH) 24:1***	↓	***3.2752***	***0.001535***	***2.8139***	***0.030619***

*Criteria selection of the most significative metabolites p<0.05 and VIP≥1. ** Arrows indicate that the relative increase/ decrease of serum level in GC as compared to FDR group. In **bold** indicated the metabolites that have passed validation step.
